# Timeline of Multi-Organ Plasma Extravasation After Bleomycin-Induced Acute Lung Injury

**DOI:** 10.3389/fphys.2022.777072

**Published:** 2022-01-31

**Authors:** Oliver Kitzerow, Irving H. Zucker, Steven J. Lisco, Han-Jun Wang

**Affiliations:** ^1^Department of Genetics Cell Biology and Anatomy, University of Nebraska Medical Center, Omaha, NE, United States; ^2^Deptrtment of Anesthesiology, University of Nebraska Medical Center, Omaha, NE, United States; ^3^Department of Cellular and Integrative Physiology, University of Nebraska Medical Center, Omaha, NE, United States

**Keywords:** inflammation, ARDS, COVID-19, bleomycin, multi-organ failure

## Abstract

Acute lung injury (ALI) is characterized by the abrupt onset of clinically significant hypoxemia in the context of non-hydrostatic pulmonary edema. Acute lung injury is associated with cytokine release and plasma extravasation (PEx) that can cause pulmonary edema and subsequently acute respiratory distress syndrome (ARDS). Therefore, it is critical we understand the relationship between ALI and lung PEx. In addition, it is also important to assess PEx in the lungs and other organs post-ALI since ALI/ARDS often causes multi-organ failure. We hypothesized that ALI induces time-dependent lung PEx, which promotes extravasation in the heart, liver, kidney, spleen, pancreas, and gastrointestinal (GI) tract, in a time-dependent manner. To test our hypothesis, we administered bleomycin or saline via tracheal intubation in 8-week-old Sprague Dawley rats. At the terminal experiments, Evans Blue was injected (IV) through the femoral vein to allow for the visualization of PEx. Plasma extravasation of desired organs was evaluated at 3-, 7-, 14-, 21-, and 28-days after bleomycin or saline treatment by evaluating Evans Blue concentrations calorimetrically at fluorescence excitation wavelength of 620 nm (bandwidth 10 nm) and an emission wavelength of 680 nm (bandwidth 40 nm). Data show that ALI induces lung PEx beginning at day 3 and peaking between 7 and 21 days. Extravasation was also seen in all organs at varying degrees beginning at day 3 and peaking between days 7 and 14. Resolution appears to start after day 21 and continues past day 28. We conclude that ALI caused by bleomycin incites a time-dependent PEx of the lungs and multiple other organs.

## Introduction

Acute lung injury/respiratory distress syndrome (ALI/ARDS) has an incidence of 200,000 per year and a high overall mortality rate of 40% in the United States (Goss et al., 2003; MacCallum and Evans, 2005; Matthay and Zimmerman, 2005; Rubenfeld et al., 2005; Bellani et al., 2016). These rates are based on the Berlin Definition, although descriptive, it is estimated to underrecognize ARDS cases (Goss et al., 2003; MacCallum and Evans, 2005; Matthay and Zimmerman, 2005; Bellani et al., 2016). In addition, clinician recognition is low with 40% of all cases being undiagnosed (Bellani et al., 2016). Even with undervalued rates, ALI/ARDS remains clinically significant and warrants further investigation.

Acute lung injury is characterized by the abrupt onset of clinically significant hypoxemia with the presence of non-hydrostatic pulmonary edema. The key pathologic influencers of ALI/ARDS being dysregulated inflammation, increased activity of leukocytes, uncontrolled activation of coagulation pathways, and altered permeability of alveolar endothelial and epithelial barriers (Butt et al., 2016; Cutts et al., 2017; D’Alessio, 2018; Nanchal and Truwit, 2018; Huppert et al., 2019; Matthay et al., 2019; Mowery et al., 2020; Wood et al., 2020). Furthermore, ALI/ARDS is often associated with multiple organ failure (Butt et al., 2016; Cutts et al., 2017; D’Alessio, 2018; Nanchal and Truwit, 2018; Huppert et al., 2019; Matthay et al., 2019; Mowery et al., 2020; Wood et al., 2020). Although the pathogenesis of ALI/ARDS has been widely studied, little is known about how it induces multiorgan failure, which we believe is caused, in part, by hypoxemia induced by pulmonary inflammation and edema.

Inflammation is an adaptive response that is triggered by noxious stimuli and conditions, such as infection and tissue injury. It is generally thought that a controlled inflammatory response is beneficial; however, it can become harmful if dysregulated. Most forms of ALI/ARDS are associated with acute cytokine release. The main and most immediate effect of these cytokines is to elicit the extravasation of plasma proteins and leukocytes that are normally restricted to the intravascular space in both lung and other organs. In the lung, this can cause pulmonary edema and lung injury. It is known that increased permeability of microvascular barriers and resultant edema is a cardinal pathophysiologic mechanism in ALI/ARDS (Huppert et al., 2019). What remains to be elucidated is the effects of pulmonary edema on organs other than the lungs. Furthermore, the time course of multiorgan inflammation after ALI/ARDS has not been investigated. To estimate the extent of multiorgan inflammation after ALI, we generated a lung injury model by instilling Bleomycin (Bleo) intra-tracheally. Evans Blue, a dye that binds albumin, was used to quantify plasma extravasation. The goals of this study were to quantify the levels of plasma extravasation (PEx) in various organs at multiple timepoints after the induction of ALI. We hypothesized that ALI induces time-dependent lung PEx, which promotes extravasation in the heart, liver, kidney, spleen, pancreas, and gastrointestinal (GI) tract, in a time-dependent manner.

## Materials and Methods

All animal experimentation was approved by the Institutional Animal Care and Use Committee of the University of Nebraska Medical Center and performed in accordance with the National Institutes of Health’s Guide for Use and Care of Laboratory Animals and in accordance with the ARRIVE guidelines (Kilkenny et al., 2012). Experiments were performed on adult, male, 200–250 g Sprague-Dawley rats purchased from the Charles River Laboratories (Wilmington, MA, United States). Animals were housed on-site and given a one-week acclimation period prior to experimentation. Food and water were supplied *ad libitum*, and rats were kept on 12-h light/dark cycles.

### Rat Model of Lung Injury

Rats were randomized into two groups: sham rats and Bleo-exposed rats. They were then randomly selected for PEx evaluation at 3-, 7-, 14-, 21-, and 28-days post-instillation. Bleo (2.5 mg/kg, ∼0.15 ml) was instilled intra-tracheally to the lungs under 3% isoflurane anesthesia. Sham control rats underwent intra-tracheal instillation of saline.

### Tissue Plasma Extravasation and Quantification of Evans Blue

Rats were anaesthetized with urethane (800 mg/kg ip) and α-chloralose (40 mg/kg ip). Evans Blue, 20 mg/kg (10 mg/ml, dissolved in heparinized saline) was administered intravenously through the femoral vein. After 10 min, rats were euthanized by transcardiac perfusion with Phosphate Buffered Saline (0.01 M, pH 7.4). Organ tissues including lungs, heart, kidney, liver, pancreas, duodenum, and spleen were excised and photographed. Then, these tissues were weighed, placed in 2 ml of N,N′-dimethyl formamide, homogenized, and incubated at 50°C water bath for 24 h. Organ tissues were then centrifuged (1 min, 14,000 rpm) and the Evans Blue content in the supernatant was determined in a 96-well microplate reader (infinite M200, TECAN, Männedorf, Switzerland) using a fluorescence excitation wavelength of 620 nm (bandwidth 10 nm) and an emission wavelength of 680 nm (bandwidth 40 nm) (100 μl sample per well). Extravasation of Evans Blue was expressed as mg Evans Blue/g of lung tissue, by comparing the experimental values with a known standard.

### Arterial Blood Gas Analysis

The artery on the ventral aspect of the rat tail was used for the collection of small amounts of blood (∼0.1 ml) for analyzing arterial blood gas at day 7 and 14 post bleomycin. The animal was restrained with a commercial restrainer so that its tail was accessible. The tail was prepared aseptically by alternating alcohol prep pads and iodine prep pads three times and the artery was then punctured using a 24 G needle. A small volume of blood (∼0.1 ml) was gently aspirated into the syringe for blood gas analysis (iSTAT, Abbott, Chicago, IL, United States). After sample collection, the needle was removed, and a gauze swab was pressed firmly on the puncture site to stop bleeding.

## Statistics

Statistical evaluation was analyzed using GraphPad Prism (GraphPad Software, San Diego, CA, United States. Version 8). Differences between treatments were determined using a Mixed-effects model for repeated-measures ANOVA. For comparison between two groups (Sham and Bleo) both Tukey and Bonferroni corrections for multiple comparisons were used with *P* < 0.05 being statistically significant.

## Results

### Evaluation of Lung Injury

**FIGURE 1:**
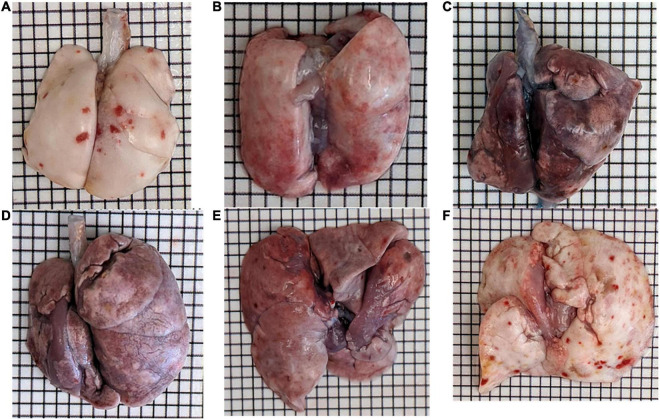
Bleo instillation induces plasma extravasation (PEx) visualized by the tissue content of Evans Blue. PEx begins to occur around day 3 **(B)** and peaks around day 7 **(C)**. PEx subsequently plateaus at day 14 **(D)** and begins to resolve at day 21 **(E)** with resolution seen at day 28 **(F)**. Day 7 control is shown in panel **(A)**.

**FIGURE 2:**
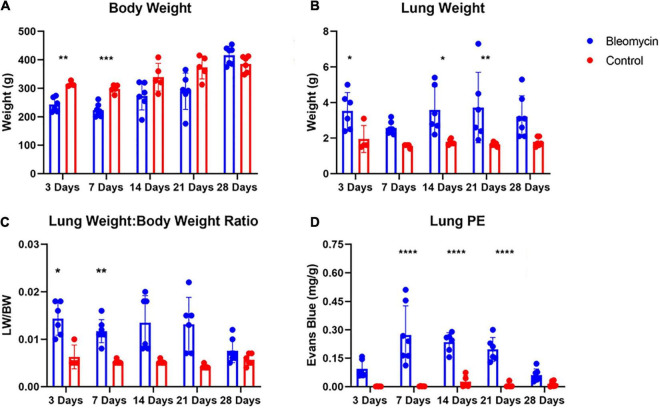
Body weight **(A)** and lung weight **(B)** for Bleo and sham rats **(A)**. Lung edema was indirectly estimated by obtaining lung weight and body weight ratio **(C)**. Bleo induces plasma extravasation within the lung at 7-, 14-, and 21-days **(D)**. Mean ± SD. *n* = 5–7 per group. Comparing bleomycin with control group *P < 0.05, **P < 0.005, ***P < 0.0005, ****P < 0.0001.

Bleomycin-treated rats exhibited lower body weights during the first week post Bleo instillation (Figure 1A). Lung weight for Bleo rats was significantly higher at 3-, 14-, and 21-days compared to the saline-treated rats (Figure 1B). Despite the lack of significance of lung weight at day 7, higher lung weight to body weight ratios were observed during the first week post-Bleo instillation (Figure 1C). Bleomycin-treated rats exhibited increased levels of PEx as signified by the levels of Evans Blue (Figure 1D). Plasma extravasation appears to increase slightly at 3 days and peak at 7 days and continue through day 21 (Figure 1E). Subsequently, at approximately day 28, PEx diminished indicating that resolution was taking place (Figure 1F). Lung visualization recapitulates the quantified data (Figures 2A–D). These data reconfirm that Bleo induces ALI/ARDS and that lung PEx initiation and resolution is time dependent.

### Evaluation of ALI Effects on Arterial Blood Gas Parameters

**FIGURE 3:**
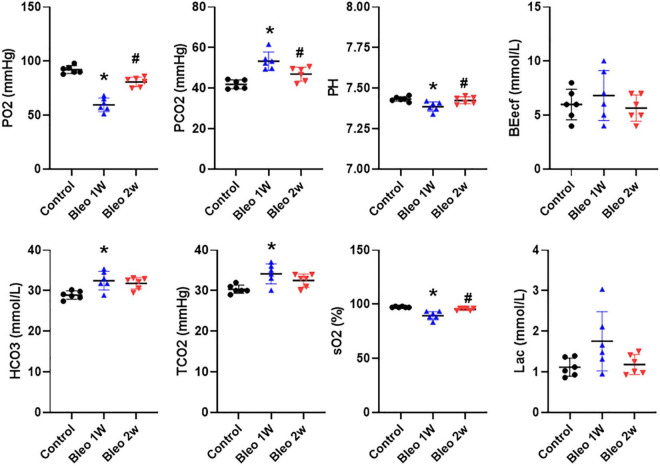
Arterial blood gases in control, 1-week and 2-week post-Bleo rats. pCO_2_, partial pressure of carbon dioxide; pO_2_, partial pressure of oxygen; BEecf, base excess; TCO_2_, total CO_2_; sO_2_, Oxygen saturation; Lac, lactate. Values are mean ± SD. *n* = 6 per group. *P < 0.05 vs. control. ^#^*P* < 0.05 vs. Bleo 1w.

In order to assess the degree of tissue hypoxia post lung injury, arterial blood gas data were evaluated in rats treated with vehicle vs Bleo. As shown in Figure 3, compared to vehicle-treated rats, Bleo rats exhibited significantly decreased blood pO_2_ (less than 60 mmHg) and sO_2_ at week 1 post Bleo, indicating severe hypoxemia during acute lung injury. Bleo rats also exhibited elevated pCO_2_ and decreased pH at week 1 post Bleo, indicating blood acidosis. At week 2 post Bleo, all parameters recovered close to the normal level (pO_2_ > 80 mmHg).

### Evaluation of ALI Effects on Organ Weight

**FIGURE 4:**
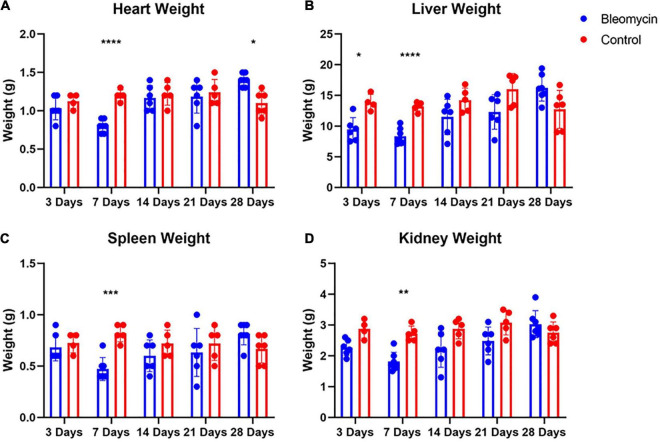
Bleo reduced organ weights at 7-days **(A–D)**. Liver weight was reduced at day 3 **(B)**. Heart weight was increased 28 days after Bleo **(A)**. Mean ± SD. *n* = 5–7 per group. Comparing Bleo with control group *P < 0.05, **P < 0.005, ***P < 0.0005, ****P < 0.0001.

Contrary to lung weight differences, Bleo rats exhibited decreased heart weight as well as a decrease in liver, spleen, and kidney weight at 7 days compared to the saline-treated rats (Figures 4A–D). In addition, the weight of the liver in Bleo rats was significantly less than saline at 3 days (Figure 4B). Surprisingly, the hearts of Bleo-treated rats weighed more than the saline-treated rats, which may indicate the beginning stages of compensatory cardiac hypertrophy (Figure 4A). These data provide evidence that ALI/ARDS causes multi-organ pathology.

### Evaluation of ALI Effects on Plasma Extravasation in Other Organs

**FIGURE 5:**
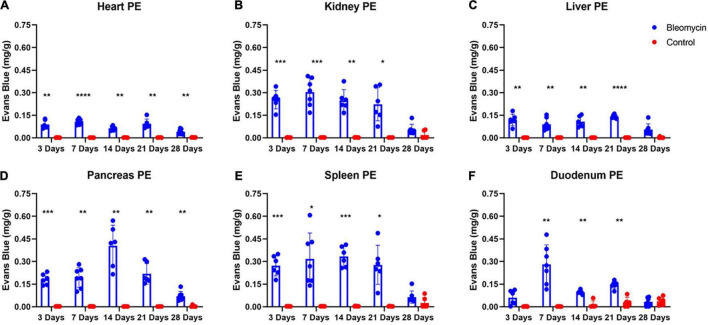
Bleo instillation caused plasma extravasation starting at day 3 for all organs except the duodenum **(A–F)**. Resolution of plasma extravasation occurred after day 21 for all organs except the heart and pancreas **(A–F)**. Mean ± SD. *n* = 5–7 per group. Comparing bleomycin with control group *P < 0.05, **P < 0.005, ***P < 0.0005, ****P < 0.0001.

Significant increases in PEx were seen in the Bleo-treated other organs compared to those in saline-treated rats. Plasma extravasation in most organs started at day 3 and peaked at day 7 (Figures 5A–E). Plasma extravasation in the pancreas peaked at day 14.

Plasma extravasation in the heart and liver was comparatively lower compared to the lungs and other organs (Figures 5A–E), indicating that these two organs were spared or shielded from the effects of ALI/ARDS. However, unlike the liver, the heart still experienced heightened levels of PEx at day 28. Pancreatic PEx was akin to cardiac PEx in that increased levels were seen early and continued past day 28, suggesting ALI may have chronic effects on these tissues.

The duodenum also experienced distinctive PEx alterations. Like the lung, PEx in the duodenum moderately increased in day 3 and was not statistically different between sham and Bleo rats until day 7 (Figures 5A–E). Afterward, duodenum PEx decreased and was comparatively low from 14 to 21 days. Like PEx in many of the other organs, duodenum PEx resolved after 28 days.

Plasma extravasation of the kidney and spleen occur faster and more profoundly than that of the lung (Figures 5A–E). However, in similar fashion to the lung, PEx of the kidney and spleen was maintained after their initial spikes and resolved at day 28.

## Discussion

In this study we found that ALI/ARDS induces time dependent PEx in both lung and other organs. To our knowledge, there is a wealth of existing literature focusing on the mechanistic inflammatory/tissue injury pathways in individual organs after acute lung injury (Izbicki et al., 2002; Chaudhary et al., 2006; Tanjore et al., 2012; Gouda and Bhandary, 2019). However, a systemic review of time-dependent PEx in major organs post lung injury has never been examined. PEx is an integrative consequence triggered by tissue hypoxia/injury, which promotes immune cell infiltration and tissue inflammation. We believe that PEx may be an early biomarker to assess the severity of organ damage and to predict the level of tissue inflammation after ALI/ARDS. The second novel concept in our study is the discovery of organ-specific PEx after lung injury. We surprisingly found that the heart and liver had less PEx compared to other organs after lung injury. Variability in individual organs may suggest organ-specific vulnerability in response to the stress of lung injury. Finally, we also report differentiation in the peak PEx time course among all organs, a characteristic that has not been reported previously.

Acute respiratory distress syndrome is a common and deadly condition in the United States (Goss et al., 2003; MacCallum and Evans, 2005; Matthay and Zimmerman, 2005; Rubenfeld et al., 2005; Bellani et al., 2016). Investigating the mechanisms of ARDS is more important than ever due to the COVID-19 pandemic; however, ARDS can be caused by pneumonia (bacterial or viral), sepsis due to non-pulmonary infections, aspiration of gastric contents, major trauma, acute pancreatitis, transfusions, drug reactions, and fungal and parasitic lung infections (Butt et al., 2016; Cutts et al., 2017; Dostalova and Dostal, 2019; Huppert et al., 2019; Matthay et al., 2019). In addition, ARDS has been shown to impact extrapulmonary organs/tissues and induce hyperinflammation, which can lead to multiorgan failure (Butt et al., 2016; Cutts et al., 2017; Dostalova and Dostal, 2019; Huppert et al., 2019; Matthay et al., 2019).

When patients die from multiorgan failure, the cause of death is often respiratory distress irrespective of the cause of the multiorgan failure (Dostalova and Dostal, 2019; Huppert et al., 2019; Matthay et al., 2019). Pulmonary fluid management combined with mechanical ventilation is considered the standard of treatment for patients experiencing respiratory distress (Matthay and Zimmerman, 2005; Bellani et al., 2016; Nanchal and Truwit, 2018; Mowery et al., 2020). Pulmonary edema, preceded by plasma extravasation, can result from a variety of modalities including direct damage to tissue or endothelial cells, degranulation of mast cells, or from sensory afferent stimulation (Skaper et al., 2014; Butt et al., 2016; Coppola et al., 2019; Huppert et al., 2019). Plasma extravasation is of particular importance when it pertains to the lungs because excessive fluids can interfere with blood-gas exchange (Knudsen et al., 2018; Coppola et al., 2019). During the first stage of ARDS—the exudative phase—diffuse alveolar damage and edema formation occurs in the alveolus and alveolar interstitium, which ultimately results in severe hypoxemia and reduced lung compliance. Our blood gas analysis data suggested that severe hypoxemia, acidosis and hypercapnia occurred in day 7 post Bleo, which is consistent with peak PEx time window post Bleo. Bleo rats largely recovered from hypoxemia and hypercapnia at day 14 post Bleo whereas PEx remained significantly elevated until day 21, indicating that PEx might be more sensitive to detect subtle changes in lung pathology than blood gas analysis.

Increased permeability also results in released cytokines entering the circulation (Bhatia and Moochhala, 2004). Cytokines and other inflammatory mediators, such as tumor necrosis factor-α, interleukin-1β, and platelet activating factor can then induce a systemic inflammatory response and/or amplify the proinflammatory response already taking place within the lung (Bhatia and Moochhala, 2004). Lung PEx data from the current study are consistent with previous studies showing that 3 days after intratracheal instillation of Bleo, only a mild increase in lung inflammation, which is a consequence of PEx, was observed (Izbicki et al., 2002; D’Alessio, 2018). Based on a report by Chaudhary et al. (2006), the levels of IL1α, IL1β, IL6, and IFNγ in lung were only elevated during days 3–9 post bleomycin and returned to normal at day 14. In addition, Izbicki et al. (2002) reported lung fibrosis peaks at day 14 and no further increases occurred through 21. However, our data showed that peak PEx occurring at day 7 and continuing through day 21, suggesting that the inflammatory cascade may take longer than what previous literature has reported (Izbicki et al., 2002; Chaudhary et al., 2006).

Patients suffering from ARDS also appear to experience extra-pulmonary manifestations including cardiovascular complications. For example, patients with lung resection often exhibit cardiac arrhythmias including atrial fibrillation and ventricular arrhythmias (Garner et al., 2017). However, the mechanisms underlying ALI-evoked cardiac arrhythmias remains unclear. A recent study from this laboratory (Hong et al., 2021) demonstrated that Bleo-induced ALI evoked dramatic increases in premature ventricular contraction (PVC) without significant cardiac inflammation and myocyte damage, suggesting that ALI-induced cardiac ventricular arrhythmia might be neurogenic. Our current PEx data further supports this concept. Compared to other organs, the heart exhibited very mild PEx throughout the ALI process. These data suggest that the heart may be more resistant to PEx when ALI occurs.

Surprisingly, the liver showed low levels of PEx, similar to that in the heart. In fact, liver function is increasingly recognized as a critical determinant of the pathogenesis and resolution of ARDS (Fuhrmann et al., 2006; Dong et al., 2020). However, the mechanisms by which ARDS impacts liver function are not fully understood. A better understanding of the lung—liver crosstalk can provide valuable insights into alternative therapeutic strategies for clinical intervention in patients with ARDS. Our current data indicated that ALI did not directly induce significant local liver inflammation since liver PEx was mild. Other factors such as hypoxia and systemic cytokine storm triggered by ALI more likely contribute to the liver dysfunction post ALI/ARDS.

Acute respiratory failure is also associated with pancreatitis with up to sixty percent of deaths within the first 7 days of acute pancreatitis being caused by acute respiratory failure (Zhou et al., 2010; Akbarshahi et al., 2012; Zhu et al., 2018). The effects of acute pancreatitis on ALI/ARDS have been studied extensively, but the influences of ALI/ARDS on acute pancreatitis requires further investigation. Our data show that PEx within the pancreas peaks at day 14, which is notably after peak lung PEx, suggesting the pancreas may be more susceptible to hypoxemia resulting from lung edema. Alternatively, peak pancreatic PEx occurs after peak PEx of the duodenum which, due to anatomical proximity, could influence pancreatic PEx.

In ARDS, the pulmonary microbiome has been found to be enriched with gut-associated bacteria, primarily represented by Bacteroidetes and Enterobacteriaceae (Dickson et al., 2016). Recent studies in sepsis, ARDS, and stroke suggest that during these conditions, certain gut microbes might increasingly translocate across the bowel wall and enter the lung (Wang et al., 2013; Bischoff et al., 2014; Dickson et al., 2016; Mukherjee and Hanidziar, 2018). Such mechanism of bacterial entry to the lung is believed to be facilitated by increased gut and alveolar permeability (Wang et al., 2013; Bischoff et al., 2014; Dickson et al., 2016; Mukherjee and Hanidziar, 2018). Increased gut permeability may also explain the subsequent peak in pancreatic PEx. In the current study we demonstrate that GI permeability increases after ALI/ARDS. If translocation of gut bacteria to the lungs occurs, it would represent an additional mechanism that potentially contributes to lung injury. It remains to be determined whether these potential pathogens, when present in the lungs of patients at risk for ARDS, are associated with adverse outcomes and therefore need to be identified early and targeted therapeutically. Our data show the highest level of PEx within the duodenum occurs at 7 days. Because increased permeability causes PEx, it is reasonable to assume that permeability is at its greatest then, and translocation of the gut microbiome should be monitored.

Acute Lung Injury/ARDS is often associated with acute kidney injury (AKI) with over 60% of ARDS patients developing AKI (Ricksten et al., 2013; Husain-Syed et al., 2016). The kidney has a high rate of oxygen consumption per gram of tissue, making the kidneys extremely sensitive to hypoxic injury (Ricksten et al., 2013). Our data show renal PEx occurring at day 3, indicating some early renal inflammation/injury. This early renal PEx could be due to lung injury-induced hypoxia.

The spleen showed similar PEx as the kidney. In humans blood travels via an open circulation through the venous sinuses of the red pulp. The endothelial cells of the sinusoids do not have characteristic cellular junctions to adjacent cells. Due to the lack of junctions, there are wide gaps between the cells that serve as a mechanical filter between the blood and splenic cords, which is a mechanism by which the spleen removes microorganisms, cellular debris, and aged and damaged erythrocytes from the circulation. However, in rodents, blood enters directly into venous sinuses making it a closed system which may be the reason for low PEx levels in saline-treated rats. Circulating immune complexes as well as autoantibodies against lung epithelial antigens have been reported in blood and bronchoalveolar lavage fluid indicating that immune responses occur distal to the lung (Liu et al., 2019; Wong et al., 2019). Because of the spleen’s immune functions, it is not surprising that PEx occurs after ALI. Our data suggest that the spleen may be mounting an immune response as soon as day 3 and continuing the response through day 21.

## Limitations

We must acknowledge a few limitations in this study. First, we only assessed PEx rather than direct measurements of inflammation and barrier disruption. The time course of the pulmonary inflammatory cascade has been well characterized in the Bleo rat model (Chaudhary et al., 2006). Based on the report by Chaudhary et al. (2006), the levels of pulmonary proinflammatory cytokines including IL1α, IL1β, IL6, and IFNγ measured by the ELISA assays were only elevated for 9 days post Bleo. However, our data indicated that PEx in the lung lasted at least 21 days post lung injury, suggesting that PEx could be a more sensitive indicator to tissue injury. We believe that PEx could provide additional valuable information independent of cytokine measurement. We also acknowledged that tissue histology such as H&E staining would have been useful in determining how plasma extravasation led to organ injury across time points. Second, we did not weigh intact GI organs because most of the GI organs still included food and stool inside the lumen. Third, Bleo can be administered intratracheally, initiating injury at the epithelium; or administered intravenously, first affecting the vascular endothelium, or intraperitoneally, which also produces systemic effects. Intratracheal instillation will first be confined to the lung, with effects then propagated to other organs. In our model, a time difference in involvement of extra-pulmonary tissues would be expected. Unfortunately, we did not detect a separation in the peak PEx time window. A potential explanation could be due to the severity of our bleomycin model. Clinically, multiple organ failure associates with end stage severe lung injury or ARDS. When modeled in animals using Bleo, lung fibrosis is typically induced by intratracheal instillation of Bleo at the dose of 1.5 mg (Kara et al., 2010), which is more than double that used in this study (1.5 mg versus 0.5–0.6 mg). Thus, the current study provides evidence that moderate lung injury is accompanied by involvement of other organs. It is possible that the dose of 1.5 mg could cause more severe ALI or ARDS, which may allow us to observe the peak PEx time window separation between lung and other organs. However, this higher dose will also cause significant mortality, impairing our primary goal to investigate the PEx time course in both acute and chronic phases of lung injury. In fact, our blood gas data, indicated that pO_2_ at week 1 post intratracheal instillation of 0.5–0.6 mg Bleo was quite severe, which should be sufficient enough to cause significant tissue hypoxia. We believe that our current Bleo dose is appropriate for the time-course study design.

## Conclusion

These data provide evidence that ALI/ARDS induced by Bleo causes time-dependent PEx in both lung and other organs, specifically the heart, kidney, liver, pancreas, spleen, and duodenum. We believe that these PEx data have significant clinical implications for understanding lung pathology as well as multi-organ injury/failure post ALI/ARDS.

## Data Availability Statement

The original contributions presented in the study are included in the article/supplementary material, further inquiries can be directed to the corresponding author.

## Ethics Statement

The animal study was reviewed and approved by the Institutional Animal Care and Use Committee of the University of Nebraska Medical Center.

## Author Contributions

OK generated and analyzed data and wrote the original draft manuscript. IZ, SL, and H-JW conceptually designed the study and reviewed, edited, and finalized the manuscript. All authors contributed to the article and approved the submitted version.

## Conflict of Interest

The authors declare that the research was conducted in the absence of any commercial or financial relationships that could be construed as a potential conflict of interest.

## Publisher’s Note

All claims expressed in this article are solely those of the authors and do not necessarily represent those of their affiliated organizations, or those of the publisher, the editors and the reviewers. Any product that may be evaluated in this article, or claim that may be made by its manufacturer, is not guaranteed or endorsed by the publisher.
